# Supplementary material for: Male meiotic spindle features that efficiently segregate paired and lagging chromosomes
Source: eLife. 2020 Mar 10;9:e50988. doi: 10.7554/eLife.50988 (PMC7101234; doi:10.7554/eLife.50988)
Supplement: Transparent reporting form [file elife-50988-transrepform.pdf]

eLIFE
1st Floor
24 Hills Road
Cambridge CB2 1JP, UK
P 01223 855340
W elifesciences.org
T @elife

<*eLife's* transparent reporting form

We encourage authors to provide detailed information *within their submission* to facilitate the interpretation and replication of experiments. Authors can upload supporting documentation to indicate the use of appropriate reporting guidelines for health-related research (see EQUATOR Network), life science research (see the BioSharing Information Resource), or the ARRIVE guidelines for reporting work involving animal research. Where applicable, authors should refer to any relevant reporting standards documents in this form.

If you have any questions, please consult our Journal Policies and/or contact us: editorial@elifesciences.org.

Sample-size estimation

- You should state whether an appropriate sample size was computed when the study was being designed
- You should state the statistical method of sample size computation and any required assumptions
- If no explicit power analysis was used, you should describe how you decided what sample (replicate) size (number) to use

Please outline where this information can be found within the submission (e.g., sections or figure legends), or explain why this information doesn't apply to your submission:

We did not use a statistical method to estimate appropriate sample sizes. We recorded and analyzed 31 spindles in meiosis I and 50 spindles in meiosis II within 6 *C. elegans* males. The total number of spindles used in the different analyses is stated both in the main text and in the corresponding figures/figure legends. For the EM analyses, we analyzed 8 complete and 3 partial spindles from 8 individual *C. elegans* males.

Replicates

- You should report how often each experiment was performed
- You should include a definition of biological versus technical replication
- The data obtained should be provided and sufficient information should be provided to indicate the number of independent biological and/or technical replicates
- If you encountered any outliers, you should describe how these were handled
- Criteria for exclusion/inclusion of data should be clearly stated
- High-throughput sequence data should be uploaded before submission, with a private link for reviewers provided (these are available from both GEO and ArrayExpress)

Please outline where this information can be found within the submission (e.g., sections or figure legends), or explain why this information doesn't apply to your submission:

The number of spindles analyzed for each experiment is shown in figure panels: 2B, 6F, 7E-F, Appendix-Figures 3, and 7.
The number of microtubules used in the comparison of different stages of cell division is given in figure panels: 6D, 8B, 8D, 8F, Appendix-Figures 5A-I, and 6B.

eLife Sciences Publications, Ltd is a limited liability non-profit non-stock corporation incorporated in the State of Delaware, USA, with company number 5030732, and is registered in the UK with company number FC030576 and branch number BR015634 at the address 1st Floor, 24 Hills Road, Cambridge CB2 1JP | August 2014

Statistical reporting

- Statistical analysis methods should be described and justified
- Raw data should be presented in figures whenever informative to do so (typically when N per group is less than 10)
- For each experiment, you should identify the statistical tests used, exact values of N, definitions of center, methods of multiple test correction, and dispersion and precision measures (e.g., mean, median, SD, SEM, confidence intervals; and, for the major substantive results, a measure of effect size (e.g., Pearson's r, Cohen's d)
- Report exact p-values wherever possible alongside the summary statistics and 95% confidence intervals. These should be reported for all key questions and not only when the p-value is less than 0.05.

Please outline where this information can be found within the submission (e.g., sections or figure legends), or explain why this information doesn't apply to your submission:

Data were plotted giving the mean value with standard deviation in all cases (shown either as error bars or shaded areas). In some plots the individual data points are included to illustrate the distribution of the data.
Distributions of individual microtubule populations were compared using a one-way ANOVA. Levels of significance shown are: * $p <= 0.05$; ** $p <= 0.01$; *** $p <= 0.001$.
In Figure 8B, D and F, the comparisons among the data sets were omitted in order to maintain the 'readability' of the graphs. All comparisons for these distributions are shown in Appendix-Figure 5.

(For large datasets, or papers with a very large number of statistical tests, you may upload a single table file with tests, Ns, etc., with reference to sections in the manuscript.)

Group allocation

- Indicate how samples were allocated into experimental groups (in the case of clinical studies, please specify allocation to treatment method); if randomization was used, please also state if restricted randomization was applied
- Indicate if masking was used during group allocation, data collection and/or data analysis

Please outline where this information can be found within the submission (e.g., sections or figure legends), or explain why this information doesn't apply to your submission:

Wild-type spindles were grouped according to their respective meiotic division (either meiosis I or II), and mutant samples were grouped according to their respective genotype (only meiosis I was investigated).
As given in Materials and Methods, all microtubules positioned within a ribosome-free zone (i.e. with a distance of max. 150 nm from the chromosome surface) were classified as either 'end-on' or 'lateral'. Then microtubules were grouped according to the closest chromosome. X chromosome-associated microtubules were assigned separately.

Additional data files ("source data")

- We encourage you to upload relevant additional data files, such as numerical data that are represented as a graph in a figure, or as a summary table
- Where provided, these should be in the most useful format, and they can be uploaded as "Source data" files linked to a main figure or table
- Include model definition files including the full list of parameters used

- Include code used for data analysis (e.g., R, MatLab)
- Avoid stating that data files are “available upon request”

Please indicate the figures or tables for which source data files have been provided:

The EM model files for microtubules and chromosome surfaces (Amira file format) have been uploaded to the TU Dresden Open Access Repository and Archive (OpARA) and are available for download as open access under:
http://dx.doi.org/10.25532/OPARA-56
